# Shear wave elasticity by tracing total nodule showed high reproducibility and concordance with fibrosis in thyroid cancer

**DOI:** 10.1186/s12885-019-6437-z

**Published:** 2020-02-12

**Authors:** Myung Hi Yoo, Hye Jeong Kim, In Ho Choi, Suyeon Park, Sang Jin Kim, Hyeong Kyu Park, Dong Won Byun, Kyoil Suh

**Affiliations:** 1 Division of Endocrinology and Metabolism, Department of Internal Medicine, Soonchunhyang University Hospital, Soonchunhyang University College of Medicine, 59 Daesagwan-ro, Yongsan-gu, Seoul, 140-743 Republic of Korea; 2 Elim Thyroid Clinic, Seoul, South Korea; 3 Department of Pathology, Soonchunhyang University Hospital, Soonchunhyang University College of Medicine, Seoul, South Korea; 4 Department of Biostatistics, Soonchunhyang University Hospital, Soonchunhyang University College of Medicine, Seoul, South Korea; 5 Division of Endocrinology and Metabolism, Department of Internal Medicine, Soonchunhyang University Cheonan Hospital, Soonchunhyang University College of Medicine, Cheonan, South Korea

**Keywords:** Elastography, Shear wave, Thyroid cancer, Fibrosis

## Abstract

**Background:**

Although shear wave elastography (SWE) is reported to be useful in detecting malignant thyroid nodules, it shows a wide range of cut-off values of elasticity index (EI) in detecting malignant nodules. The cause of discrepancy remains unclear. Fibrosis of the tumors is known to increase the EI in SWE, and matching of SWE and surgical histopathology has not been fully illustrated in thyroid cancer. We aimed to evaluate the reproducibility of the new total nodular region of interest (ROI) method excluding the subjective features of focal circular ROI placement and to determine the lesion that causes the elevation of EI on SWE and on histopathology.

**Methods:**

A total of 29 thyroid cancers from 28 patients were included. We evaluated the reproducibility of EI in the new total nodular ROI using Q-Box Trace program and compared the EI in focal nodular ROI using a 3-mm circular area. We analyzed the correlation between fibrosis and EI.

**Result:**

The coefficient of variation (CV) of the intrarater assay was significantly lower in total nodular ROI than in focal nodular ROI within the image in rater 1 (1.7% vs. 13.4%, *p* < 0.001) and in rater 2 (1.4% vs. 16.9%, *p* < 0.001) and in different images in rater 1 (7.6% vs. 12.3%, *p* = 0.040) and in rater 2 (7.5% vs. 19.8%, *p* = 0.004). Moreover, CV of the interrater assay showed similar results (14.9% vs. 19%, *p* = 0.030). Interrater intraclass correlation coefficient showed better agreement in total nodular ROI than in focal nodular ROI (0.863 vs. 0.783). The degree of fibrosis on histopathology showed significant correlations with EI (E_Mean_, *p* < 0.001; E_Max_, *p* = 0.027), and the location of fibrosis was concordant with the high EI area on SWE.

**Conclusion:**

Our study revealed that the new total nodular ROI method showed higher reproducibility and better agreement in intra- and interrater assay than the focal nodular ROI method, suggesting a valuable and standardized method in clinical practice. Moreover, our results showed that fibrosis in the histopathology increased EI on SWE and might lead to the discrepancy of the cut-off values in detecting thyroid cancer.

## Background

A thyroid nodule is a common finding in up to 60% of the population during ultrasound (US) examination, and the malignancy rate for the thyroid nodules is 5–15% [1, 2]. Fine-needle aspiration (FNA) cytology is the first step that is performed to differentiate malignant nodules; however, 5–15% of FNA revealed inadequate nondiagnostic samples, and 15–30% of FNA result in indeterminate cytologic findings category III (atypia or follicular neoplasm of undetermined significance) and category IV (suspicious for follicular neoplasm) according to the Bethesda system [3, 4]. Hence, additional tools to aid in the differentiation of malignant nodules are needed.

US elastography had been reported to be useful in the differentiation of benign and malignant thyroid nodules [5–9]. Strain elastography was initially developed with the operator using manual compression on the tissue to measure tissue displacement (strain) caused by the compression (stress) [10]. However, strain elastography had several disadvantages including high operator dependence in terms of compression and absence of sufficient quantitative information [11, 12]. Shear wave elastography (SWE) uses several focused ultrasonic pushing beams to generate shear waves and measures transversely propagated shear wave speed, and the subsequent ultrafast echographic imaging sequence generates a quantitative elastogram [13]. SWE has sufficient quantitative information and is operator independent in terms of compression; hence, it is expected to result in more reproducible findings than strain elastography, and two-dimensional SWE (2D-SWE) represents focal tissue stiffness map [14, 15]. Studies using SWE to assess the thyroid nodules have reported its usefulness in detecting malignant nodules. However, there was a wide range of cut-off values of elasticity index (EI) in detecting malignant nodules ranging from 34 kPa to 90 kPa [9, 16–25]. Thyroid nodules usually show heterogeneous images of EI within the nodule on 2D-SWE; thus, selecting different locations of region of interest (ROI) within the nodule displays different ROI even with the same operator [12, 26]. Difficulty in imaging and the subjective features of selecting representative location of ROI in thyroid nodules with heterogeneous EI contribute to variable EI profiles in SWE [27, 28]. Therefore, SWE is not operator dependent in terms of the added stress (compression) but operator dependent in the placement of ROI. To decrease this subjective variance in the placement of ROI in thyroid nodules, we let the total nodular area the ROI by tracing the total nodular margin using the overlapping B-mode US. To the best of our knowledge, this is the first trial to evaluate the total area of the nodule by tracing the nodular margin. We compared the reproducibility and reliability of EI between the total nodular ROI and the focal nodular ROI using 3-mm circular area.

Tissue elasticity is known to increase more in fibrosis [14] than in solid tumor formation [29]. Additionally, it has been reported that elasticity on strain elastography and SWE is increased relative to fibrosis but not with cellularity in the papillary thyroid carcinoma (PTC) [30–33]. We attempted to determine the pathological findings matching the high EI areas in 2D-SWE by comparing 2D-SWE of the thyroid nodule with surgical histopathology.

First, we aimed to investigate the reliability and reproducibility of EI by SWE in the new total nodular ROI and to compare it with EI in the focal nodular ROI to validate the adequacy of EI by SWE as a reliable parameter of stiffness. Second, we examined the relationship between the degree and location of fibrosis in the surgical histopathology and the magnitude and location of high EI area on 2D-SWE.

## Methods

### Study population

Data of 588 patients who visited the thyroid clinic of the Soonchunhyang University Hospital for the evaluation of thyroid nodules and who underwent SWE before US guided FNA or core-needle biopsy from November 2015 to May 2018 were retrospectively reviewed for inclusion in this study.

Among these subjects, 44 were found to have thyroid cancer on surgical pathology. Patients with poor results in SWE including, thyroid nodules with poor shear wave mapping (*n* = 7) or with macrocalcifications (*n* = 3) and presence of nodules at the isthmic/paraisthmic areas due to the interference produced by the tracheal cartilage (*n* = 5) were excluded. Finally, data from 29 thyroid cancers in 28 patients were included in this study. There were 24 cases of conventional type of PTC, 2 cases of follicular variant PTC, 2 cases of follicular thyroid carcinoma and 1 case of medullary thyroid carcinoma.

### Gray-scale ultrasound and shear wave elastography examinations

The patients were positioned for US with their necks extended. Each patient underwent gray-scale US and SWE using the Aixplorer US system (SuperSonic Imagine, Aix-en-Provence, France) and a linear probe with a frequency range of 15–4 MHz. During gray-scale US examination, thyroid nodules were evaluated for size (width, depth and length), volume, composition, orientation, echogenicity, shape, margin, and presence or absence of calcification.

After the gray-scale US, SWE was performed by the same operator who had performed the gray-scale US using the same probe. To avoid compression artifacts, generous amount of gel was used. The probe was held static in the transverse plane at the center of the nodule until the image had stabilized. After two or three cine-loop images were acquired, one representative elastogram with the fewest artifacts in each cine-loop image was selected.

Total nodular ROI was determined by drawing the margin of the nodule guided by the overlaid B-mode anatomic scan with the mode of the machine setting using the Q-Box Trace program. We drew the margin of ROI avoiding the areas of macrocalcification and cystic portion. The EI of the total nodular ROI was calculated and displayed using the machine. The mean (E_Mean_), minimum (E_Min_), maximum (E_Max_), and standard deviation (E_SD_) of SWE EI values in the ROI were calculated as kPa, and 2D-SWE was color coded from dark blue (less than 36 kPa), light blue (36–72 kPa), green, yellow, to red (greater than 180 kPa). For the focal nodular ROI, the EI in 2–3 ROI using a 3-mm circular area containing the stiffest area was measured. Moreover, the high EI area of the nodule where the color-coded EI showed higher EI than light blue (which is greater than 36 kPa) was traced by the manual drawing, and the area was calculated and displayed by the machine. The percentage of high EI area was calculated as high EI area divided by the total nodular area. For the evaluation of interrater agreement, the second physician performed the same examination immediately after the first physician.

### Pathological analysis

Surgical specimen was cut in coronal section in the largest diameter corresponding to the center of the nodule on 2D-SWE. Surgical histopathology was stained with hematoxylin and eosin staining and was diagnosed according to the World Health Organization classification [34]. Collagen fibers on histological slides were stained with Masson’s trichrome stain. Under low-power microscopy (× 12 or × 40), the image of the area showing the thyroid carcinoma was taken. The area of fibrosis and the total area of the thyroid carcinoma under the low-power microscopy images were taken, and the areas were measured using the Q-Box Trace program calculating the area with the irregular margin, and the percentage of fibrotic area was calculated by dividing the fibrotic area by the total area of the tumor.

### Statistical analysis

The tumor size and elasticity values of all the lesions were expressed as medians (25th, and 75th percentile). The differences between the thyroid carcinoma groups were compared using the Mann-Whitney U-test.

To assess the reproducibility of elasticity measurement, intra- and interrater agreements were evaluated using the intraclass correlation coefficient (ICC; two-way random, absolute agreement). Agreement for interrater measurements was also analyzed using Bland-Altman plots [35–37].

The coefficient of variation (CV) was calculated as the ratio of the standard deviation and mean value. A linear regression model was used to examine the correlation between EI and the percent of high EI area on SWE with the degree of fibrosis on histopathology. All statistical analyses were performed using the SPSS Statistics 25.0 software package (Chicago, IL, USA). *P* values less than 0.05 were considered statistically significant.

## Results

### Intra- and Interrater agreement of mean elasticity in each method

One representative elastogram from each cine-loop of 2 images was selected, and a total of 80 images from the 20 thyroid nodules from 18 subjects by two raters were obtained. The mean, SD, minimum, and maximum elasticity values of E_Mean_, and intra- and interrater agreement for the quantitative measurements of E_Mean_ are summarized in Table 1. For the total nodular ROI method, there was good intrarater agreement of E_Mean_, and ICC in rater 1 and rater 2 were 0.975 and 0.999 within the image and 0.959 and 0.976 in different images, respectively. The intrarater ICC of the focal nodular ROI method in rater 1 and rater 2 were 0.866 and 0.934 within the image and 0.734 and 0.894 in different images, respectively.

**Table 1:** Shear wave elasticity values, and intra- and interrater intraclass correlation coefficient (ICC) agreement and coefficient of variation (CV) for E_Mean_ (mean elasticity) in region of interest (ROI) with the two methods (total nodular ROI with tracing total nodule and focal nodular ROI with 3 mm circle) by two raters

Method of ROI	E_Mean_ (kPa), mean ± SD (min-max)		ICC (95% CI)	CV (%)
	Read 1	Read 2		
Total nodular ROI				
Intrarater assay (within image)				
Rater 1	25.3 ± 10.0 (12.0–49.6)	24.7 ± 9.0 (12.1–40.1)	0.975 (0.983–0.990)	1.7
Rater 2	24.3 ± 11.7 (9.6–58.7)	24.3 ± 11.9 (9.2–59.9)	0.999 (0.997–1.000)	1.4
Intrarater assay (different image)				
Rater 1	24.8 ± 9.0 (12.0–40.0)	24.6 ± 9.4 (9.3–41.7)	0.959 (0.895–0.984)	7.6
Rater 2	24.3 ± 11.7 (9.6–58.7)	26.0 ± 13.1 (10.3–60.8)	0.976 (0.933–0.991)	7.5
Interrater assay	25.3 ± 10.0 (12.0–49.6)	24.3 ± 11.7 (9.6–58.7)	0.863 (0.653–0.946)	14.9
Focal nodular ROI				
Intrarater assay (within image)				
Rater 1	34.0 ± 17.2 (14.4–86.3)	39.6 ± 22.9 (16.3–115.2)	0.934 (0.842–0.973)	13.4
Rater 2	45.0 ± 36.8 (12.4–179.9)	36.6 ± 23.4 (10.4–111.8)	0.866 (0.693–0.945)	16.9
Intrarater assay (different image)				
Rater 1	34.0 ± 17.2 (14.4–86.3)	33.6 ± 11.9 (11.2–53.7)	0.894 (0.730–0.958)	12.3
Rater 2	45.0 ± 36.8 (12.4–179.9)	37.1 ± 19.1 (11.1–93.1)	0.734 (0.347–0.894)	19.8
Interrater assay	34.0 ± 17.2 (14.4–86.3)	45.0 ± 36.8 (12.4–179.9)	0.783 (0.443–0.915)	19.0

**Fig. 1:**
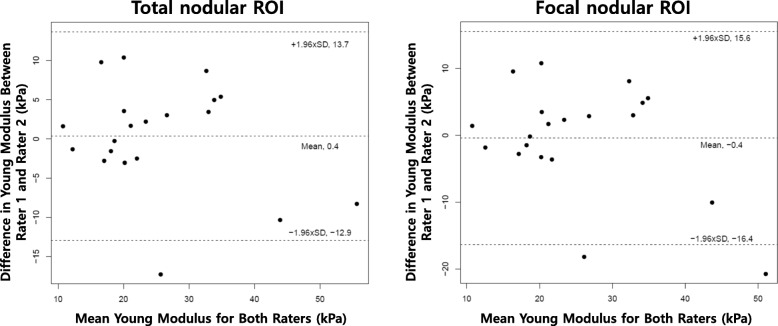
Bland-Altman plot of Young Modulus (E_Mean_) between two raters. **a** Total nodular ROI. **b** Focal nodular ROI. E_Mean_, mean elasticity; ROI, region of interest; kPa, kilo-Pascal

The first elastogram in each cine-loop was used to calculate the ICC and to assess the interrater agreement. Interrater ICC of the total nodular ROI and focal nodular ROI were 0.863 and 0.783, respectively. The intrarater CVs of the total nodular ROI method in rater 1 and rater 2 were significantly lower than those of the focal nodular ROI method in rater 1 (1.7% vs. 13.4%, *p* < 0.001) and in rater 2 (1.4% vs. 16.9%, *p* < 0.001) within the image and in rater 1 (7.6% vs. 12.3%, *p* = 0.040) and in rater 2 (7.5% vs. 19.8%, *p* = 0.004) in different images. The interrater CV of the total nodular ROI method was significantly lower than the focal nodular ROI method (14.9% vs. 19.0%, *p* = 0.030). Analysis using the Bland-Altman plots for E_Mean_ between the two raters is shown in Fig. 1. Bland-Altman analysis between the two raters for the total nodular ROI method showed a bias of 0.4 and limits of agreement of − 12.9 to 13.7, with a repeatability coefficient between the 2 raters of 13.3. The focal nodular ROI method showed a bias of − 0.4 and limits of agreement of − 16.4 to 15.6, with a repeatability coefficient between the 2 raters of 16.0.

### Correlation between elasticity index measurements on shear wave Elastography and the degree of fibrosis on surgical histopathology

**Fig. 2:**
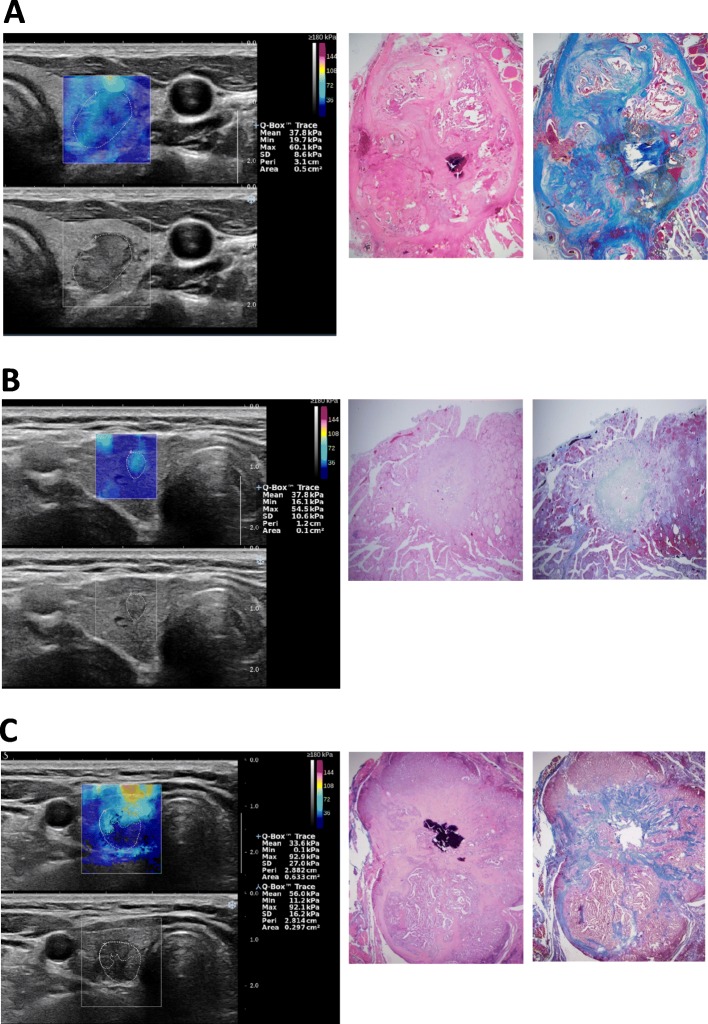
Shear wave elastography (SWE), gray-scale ultrasound (US), H&E staining, and Masson’s trichrome staining slides of the papillary thyroid carcinoma in 3 patients. **a** The case is from a patient with a 1.63-cm left middle thyroid nodule. A suspicious malignant nodule with hypoechogenicity and lobulated margin was observed on gray-scale US. SWE showed generalized uneven distribution of high elasticity index (EI) area within the nodule. Masson’s trichrome staining showed uneven irregular distribution of fibrosis within the nodule, concordant with the high EI area on SWE. **b** The case is from a patient with a 0.52-cm right middle thyroid nodule. A suspicious malignant nodule with taller-than-wide shape, microcalcification, and irregular margin was observed on gray-scale US. SWE showed high EI area over the entire nodule. On Masson Trichrome staining, fibrosis was observed in the entire nodule, concordant with the high EI area on SWE. **c** The case is from a patient with a 1.00-cm right lower thyroid nodule. A suspicious malignant nodule with hypoechogenicity, microcalcification, and lobulated margin was observed on gray-scale US. SWE showed high EI area in the upper portion of the nodule. Similar to the high EI area on SWE, fibrosis was observed in the upper portion of the nodule on Masson’s trichrome staining

**Fig. 3:**
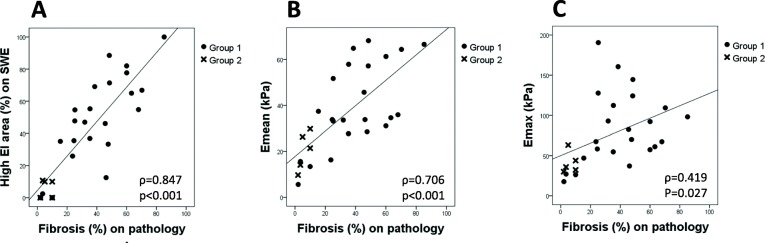
Correlation between elasticity index (EI) measurements on shear wave elastography (SWE) and the degree of fibrosis on surgical histopathology. **a** high EI area on SWE. **b** E_Mean_. **c** E_Max_. Group1, conventional type of papillary thyroid carcinoma (*n* = 24); Group 2, non-conventional papillary thyroid carcinoma including follicular variant of papillary thyroid carcinoma (*n* = 2), follicular thyroid carcinoma (*n* = 2), and medullary thyroid carcinoma (*n* = 1). E_Mean_, mean elasticity; E_Max_, maximum elasticity; kPa, kilo-Pascal

**Fig. 4:**
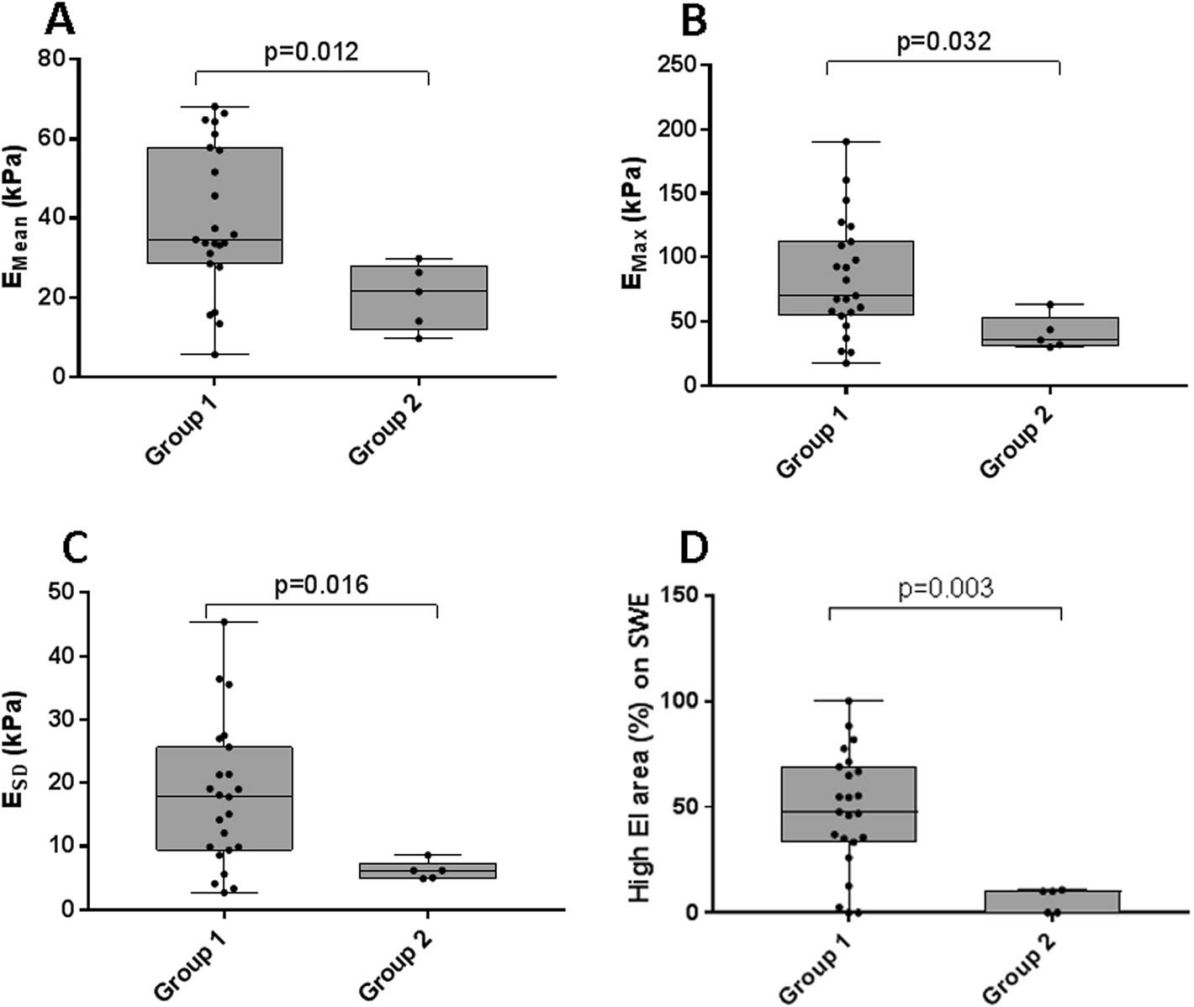
Box-and-whisker plots of elasticity index (EI) measurements on shear wave elstography (SWE) for group 1 and group 2. **a** E_Mean_. **b** E_Max_. **c** E_SD_. **d** high EI area on SWE. Group1, conventional type of papillary thyroid carcinoma (*n* = 24); Group 2, non-conventional papillary thyroid carcinoma (*n* = 5) including follicular variant of papillary thyroid carcinoma (*n* = 2), follicular thyroid carcinoma (*n* = 2), and medullary thyroid carcinoma (*n* = 1); E_Mean_, mean elasticity; E_Max_, maximum elasticity; E_SD_, one standard deviation of elastographic values; kPa, kilo-Pascal

To evaluate the correlation between EI measurements on SWE and the degree of fibrosis on surgical histopathology, we reviewed and compared the SWE images and the histological specimens for carefully selected group of patients as shown in Fig. 2. Our data suggested a strong positive correlation between high EI area on SWE and the degree of fibrosis on surgical histopathology (*r* = 0.847, *p* < 0.001) (Fig. 3a), and also between E_Mean_ and the degree of fibrosis on surgical histopathology (*r* = 0.706, *p* < 0.001) (Fig. 3b). E_Max_ showed a weak positive correlation with the degree of fibrosis on surgical histopathology (*r* = 0.419, *p* = 0.027) (Fig. 3c). The fibrosis on surgical histopathology was higher in conventional PTCs (Group 1) than in the non-conventional PTCs including follicular variant PTCs, follicular thyroid carcinomas, and a medullary thyroid carcinoma (Group 2). Group 1 showed higher EI values and EI area than Group 2, which were statistically significant for E_Mean_ (*p* = 0.012), E_Max_ (*p* = 0.032), E_SD_ (*p* = 0.016), and high EI area on SWE (*p* = 0.003) (Fig. 4).

## Discussion

SWE is operator independent in terms of adding stress but is operator dependent in the placement of ROI, and the difficulty in images and the subjective features of selecting representative location of ROI have been reported as the causes of variability of SWE [14, 27, 28].

In this study, we examined a new method of placement of ROI by tracing the total nodule and evaluated the reproducibility as well as compared total nodular ROI with conventional focal ROI using a 3-mm circular area. The intrarater CVs of the total nodular ROI method in rater 1 and rater 2 were significantly lower than those of the focal nodular ROI method in rater 1 (1.7% vs. 13.4%, *p* < 0.001) and in rater 2 (1.4% vs. 16.9%, *p* < 0.001) within the image and in rater 1 (7.6% vs. 12.3%, *p* = 0.040) and in rater 2 (7.5% vs. 19.8%, *p* = 0.004) in different images. Moreover, the interrater CV of the total nodular ROI method was significantly lower than that of the focal nodular ROI method (14.9% vs. 19.0%, *p* = 0.030). Bardet et al. [26] reported that the intra- and interobserver CVs for the mean EI in the focal nodular ROI were 23 and 26%, respectively. Brezak et al. [38] reported that the intra- and interreader CVs in the focal nodular ROI for the mean EI were 13 and 21%, respectively. Our results are compatible with those reported by these two previous studies.

Moreover, the agreement of the mean EI between the total and focal nodular area ROI was evaluated by ICC in this study. The mean EI in the total nodular ROI showed better agreement than in the focal nodular ROI in intra-assay agreement (ICC, 0.959 vs. 0.894 in rater 1 and 0.976 vs. 0.734 in rater 2) and also in interrater agreement (ICC, 0.863 vs. 0.783). Bland-Altman plot analysis of interrater agreement showed better interrater agreement of EI in the total nodular ROI than in the focal nodular ROI. Moreover, our study revealed that SWE EI measurement in the total nodular ROI was more reproducible and showed less variability compared to the mean EI in the focal nodular ROI and suggested that it may be a valuable and standardized method in clinical practice.

A comparison of the EI with the degree of fibrosis, E_Mean_ and E_Max_ showed a correlation with the percent of fibrosis. Additionally, comparison of EI with surgical histopathology revealed that high EI areas in SWE in thyroid cancer were concordant to the areas of fibrosis. PTC is commonly associated with fibrotic change particularly evident at the advancing edge [34] and up to 80% of PTC show fibrotic change with various degrees [39, 40], while stromal component of follicular neoplasm especially adenoma is typically scant [34] and occasionally shows stromal fibrosis and hemorrhage [40]. Regarding follicular thyroid carcinoma, several studies reported that SWE did not show significantly elevated elasticity [7, 20] similar to benign nodules. Rago et al. [30] reported that the elasticity of the thyroid nodules with stain elastography did not correlate with cell number, and nodule stiffness was correlated with the degree of fibrosis, which was more evident in classic PTC than in follicular variant PTC. Yi et al. [31] reported that the strain ratio of US elastography was positively correlated with the degree of fibrosis of PTC on surgical histopathology. Fukuhara et al. [32, 33] reported that when malignant thyroid nodules were classified according to the degree of fibrosis on histopathology, SWE showed high shear wave velocity in nodules with severe fibrosis and malignant nodules with no fibrosis showing similar shear wave velocity with benign nodules with no fibrosis, and the effect of cell density on shear wave velocity was insignificant. These reports suggest that fibrosis plays a major role in the elasticity of the thyroid nodules, and elevated elasticity in thyroid cancer is mainly related with fibrosis, which is frequent in PTC and non-conventional PTC with less or no fibrosis showing insignificant or slight elevation of elasticity including follicular carcinoma and follicular variant PTC [25, 26]. Although E_Mean_ has been reported to be significantly higher in conventional PTC than in non-conventional PTC, non-conventional PTC in our study was a heterogeneous group of thyroid cancers and the number was too small. Thus, large prospective studies evaluating non-conventional PTC are needed to verify our results.

Our data revealed that the percentage of high EI (greater than 36 kPa) area of the nodule showed a correlation with the degree of fibrosis (percentage of fibrosis on surgical histopathology). Moreover, EI (E_Max_ and E_Mean_) was correlated with the degree of fibrosis. Additionally, the location of fibrosis was concordant with high EI area. Hence, the degree and location of fibrosis on histopathology were closely correlated with the high EI of the thyroid nodule. Diverse elasticity in PTC may reflect various degrees of fibrosis in PTC, resulting in the discrepant cut-off values in the diagnosis of malignant thyroid nodules, because more than 80% of malignant nodules are composed of PTC. A wide range of cut-off values of EI in detecting malignancy from 34 kPa to 90 kPa has been reported [9, 16–25], and which may be due to the various degrees of fibrosis in different patients and different types of tumors in the same study and also in the different study groups.

## Conclusions

Our study revealed that the mean EI in the total nodular ROI showed higher reproducibility and better agreement in intra- and interrater assay than in the focal nodular ROI when evaluating the ICC, CV, and Bland-Altman analysis, which may be due to the multiple reasons including the avoidance of the subjective variance of placement of ROI in the focal nodular area. We suggest that the total nodular ROI method may be a valuable and standardized method in clinical practice. Our results showed that fibrosis increased SWE elasticity in the histopathology of the thyroid nodule, which might lead to the discrepancy of the cut-off values in detecting thyroid cancer. A limitation of this study is the relatively small number of patients, and future studies with larger sample sizes may be needed for further clarification of the possible characteristic features of SWE.

## Data Availability

The datasets used and analyzed in the current study are available from the corresponding author on reasonable request.

## Abbreviations

CNB: Core-needle biopsy
CV: Coefficient of variation
EI: Elasticity index
E_Max_: Maximum elasticity
E_Mean_: Mean elasticity
E_Min_: Minimum elasticity
E_SD_: One standard deviation of elastographic values
FNA: Fine-needle aspiration cytology
ICC: Intraclass correlation coefficient
PTC: Papillary thyroid carcinoma
ROI: Region of interest
SWE: Shear wave elastography
US: Ultrasound
